# The effects of visual art therapy in older adults with mild cognitive impairment: a systematic review and meta-analysis

**DOI:** 10.3389/fpubh.2026.1765620

**Published:** 2026-03-23

**Authors:** Yang Jiao, Yunxu Zhang, Jinhua Liu

**Affiliations:** 1 College of Arts, Sichuan University, Chengdu, China; 2 International School of Law and Society, Sichuan International Studies University, Chongqing, China; 3 Institute of Social Governance, Southwest Petroleum University, Chengdu, China

**Keywords:** cognitive rehabilitation, meta-analysis, mild cognitive impairment, non-pharmacological interventions, visual art therapy

## Abstract

**Objective:**

To systematically evaluate the effectiveness of visual art therapy (VAT) in older adults with mild cognitive impairment (MCI).

**Methods:**

A systematic search of multiple bibliographic databases and gray literature sources was conducted from inception to January 25, 2026, and RCTs comparing VAT with control conditions were included. Two reviewers independently screened studies, extracted data, and assessed risk of bias. Continuous outcomes were pooled using a random-effects model and summarized as SMDs or MDs. Heterogeneity was assessed using Cochran’s *Q* test and the *I*^2^ statistic. Meta-analyses were performed in RevMan 5.3, and certainty of evidence was rated using the GRADE approach.

**Results:**

Nine RCTs (*N* = 595) were included. For the primary outcome, VAT improved global cognition [SMD = 0.55, 95% CI 0.38 to 0.72; *I*^2^ = 0%; (number of studies) *k* = 8; *N* = 564, moderate certainty]. For secondary outcomes, VAT improved long-term memory (SMD = 0.34, 95% CI 0.13 to 0.56; *I*^2^ = 0%; *k* = 5; *N* = 331; low certainty) and short-term memory (SMD = 0.42, 95% CI 0.19 to 0.65; *I*^2^ = 11%; *k* = 4; *N* = 336; low certainty). No clear benefit was observed for language function (SMD = 0.14, 95% CI −0.10 to 0.37; *I*^2^ = 0%; *k* = 4; *N* = 277; low certainty). VAT reduced depressive symptoms (SMD = −0.53, 95% CI −0.86 to −0.21; *I*^2^ = 42%; *k* = 5; *N* = 312; low certainty) and improved activities of daily living (SMD = −0.41, 95% CI −0.64 to −0.19; *I*^2^ = 0%; *k* = 4; *N* = 305; low certainty).

**Conclusion:**

Current RCT evidence suggests that VAT improves global cognition in older adults with MCI and may improve memory, depressive symptoms, and activities of daily living, whereas no clear benefit was found for language function. These findings require confirmation in larger, methodologically rigorous trials with more standardized outcome measurement.

**Systematic review registration:**

https://www.crd.york.ac.uk/PROSPERO/view/CRD420251167927, CRD420251167927.

## 1 Introduction

With the rapid acceleration of global population aging, cognitive impairment has become a major clinical and public health challenge. Currently, over 55 million people worldwide are living with dementia, and this figure is projected to reach 139 million by 2050 (1). Mild cognitive impairment (MCI) is commonly viewed as a transitional stage between normal aging and dementia, characterized by mild declines in cognitive function while independence in activities of daily living is largely preserved (2). Importantly, individuals with MCI have a substantially increased risk of progression to dementia; therefore, MCI is widely regarded as a critical window for intervention and dementia prevention efforts (3–5).

Although drug development has continued to advance, current evidence remains insufficient to support a routinely recommended standard pharmacological treatment regimen. Overall, no medication has been clearly established as a routine therapy for MCI, and commonly used agents such as cholinesterase inhibitors have shown limited overall benefit at the MCI stage while being associated with frequent adverse effects (6, 7). Consequently, increasing attention has been directed toward non-pharmacological interventions that are more accessible, suitable for older adults, and scalable in community and primary care settings (8, 9).

Against this backdrop, visual art therapy (VAT) has attracted increasing attention (10–12). Compared with broader creative arts therapies (e.g., music, dance, and drama), different artistic media may differ systematically in delivery modes, active components, and potential mechanisms. VAT is grounded in visually mediated activities and typically engages visuospatial processing, attention, executive control, and fine motor coordination—functional domains that are frequently affected in the MCI impairment profile—thereby providing a stronger “media-specific” rationale for intervention (13, 14). Meanwhile, the overall evidence base for creative arts interventions is relatively more extensive, with a larger body of studies—particularly on music therapy and dance-based interventions—reporting outcomes relevant to cognitive impairment in older adults, thus offering more accumulated evidence for potential benefits (15–20). However, evidence on VAT specifically for older adults with MCI remains fragmented, and methodological quality varies across studies. For example, some studies enrolled broader populations rather than focusing exclusively on individuals with MCI (21, 22); others examined multiple forms of arts-based interventions rather than VAT *per se* (23–25). In addition, some reviews included RCTs alongside lower-quality designs such as non-randomized controlled trials (non-RCTs) (21, 22, 26), which may compromise the credibility of the synthesized evidence.

Because there is no universally accepted definition of VAT, we developed an operational definition by drawing on the British Association of Art Therapists’ definition of art therapy (27) and on prior evidence syntheses that have proposed operational criteria for active visual arts interventions (28, 29). In this study, VAT is operationally defined as a structured psychotherapy with explicit therapeutic or rehabilitative goals, in which visual art media constitute the primary therapeutic modality (e.g., painting, drawing, collage, calligraphy, sculpture, and other handicraft-based forms) and are delivered by a therapist or a trained facilitator. Notably, interventions primarily based on music, dance, or dramatic performance, or those in which visual art is not a core component (e.g., broad multimodal expressive arts), were not included. Any mention of music, dance, or expressive arts in this manuscript is for background comparison only and does not fall within the scope of eligible interventions.

Existing studies suggest that visual arts–related activities may confer benefits for individuals with MCI by promoting neuroplasticity, enhancing cognitive function, and alleviating affective symptoms (13, 14). To improve clinical interpretability and maintain consistency in outcome selection, we framed outcomes within the *International Classification of Functioning, Disability and Health* (ICF). Global cognition was prespecified as the primary outcome. Secondary outcomes included memory, language, depressive symptoms, and activities of daily living (ADL), capturing clinically relevant domains across cognition, mood, and functional status in MCI. Accordingly, this study aimed to evaluate the effects of VAT on global cognition, specific cognitive domains, depressive symptoms, and activities of daily living in older adults with MCI through a systematic review and meta-analysis of RCTs, providing a more clinically interpretable synthesis to inform both clinical practice and public health implementation.

## 2 Methods

### 2.1 Research framework

This review was reported in accordance with PRISMA 2020 (the PRISMA 2020 checklist is provided in Supplementary Material 1) (30), and the protocol was registered in PROSPERO (CRD420251167927). Eligibility criteria were developed using the PICOS framework. Outcomes were mapped to domains of the *International Classification of Functioning, Disability and Health* (ICF) (31), as summarized in Table 1.

**Table 1:** PICOS framework.

Population (P)	Intervention (I)	Comparator (C)	Outcome (O)	Study type (S)
Older adults with mild cognitive impairment (age ≥ 60).	VAT: a structured psychotherapeutic intervention with a clear therapeutic purpose, in which visual art media are the primary therapeutic modality (e.g., drawing/painting, coloring, collage, calligraphy, sculpture, pottery/other crafts), delivered by a qualified therapist or trained facilitator.	Passive controls (no intervention, usual care, or wait-list) and active controls (health education or cognitive training).	Primary: global cognition (ICF code: b117). Secondary: memory function (b144); language function (b167); depressive symptoms (b152); activities of daily living (d5).	RCTs

### 2.2 Literature search strategy

We conducted systematic searches in PubMed, PsycINFO, the Cochrane Library, Web of Science, Embase, Scopus, CINAHL, ProQuest (journal databases), and China National Knowledge Infrastructure (CNKI) from database inception to January 25, 2026. To capture gray literature, we additionally searched ClinicalTrials.gov, ProQuest Dissertations and Theses Global (PQDT Global), and the WHO International Clinical Trials Registry Platform (WHO ICTRP) over the same time frame (full search strategies for all databases are provided in Supplementary Material 2). We also performed backward citation tracking by screening the reference lists of included studies and relevant reviews to identify any eligible studies that may have been missed. To ensure the feasibility of full-text assessment and data extraction, searches were restricted to publications in English and Chinese.

Taking PubMed as an illustration: (“art therapy” OR “visual art therapy” OR “visual art” OR “visual aesthetic” OR “art intervention” OR “creative art” OR “art psychotherapy” OR “painting” OR “drawing”) AND (“cognitive dysfunction” OR “cognitive impairment” OR “mild cognitive decline” OR “MCI” OR “subjective cognitive complaint” OR “subjective memory impairment”) AND (“people over 60” OR “older adults” OR “aged” OR “geriatric”).

### 2.3 Inclusion and exclusion criteria

Inclusion criteria: (1) Population: Older adults with MCI (aged ≥60 years), diagnosed using validated cognitive assessment instruments; (2) Intervention: The experimental group received VAT. VAT had to meet the following criteria: (i) a structured psychotherapy in which visual art media were the primary therapeutic modality, including one or more of the following activities: painting, drawing, coloring, collage, calligraphy, pottery, sculpture, and handicraft-making; and (ii) a clearly specified therapeutic purpose, delivered and/or facilitated by a therapist or a professionally trained facilitator; (3) Comparators: Given the limited number of available studies and to maximize evidence coverage, we applied broad comparator criteria, including passive controls (no intervention, usual care, wait-list) and active controls (health education, cognitive training); (4) Outcomes: (i) Primary outcome—global cognitive function; (ii) Secondary outcomes—memory function, language function, depressive symptoms, and activities of daily living; (5) Study type: RCTs.

Exclusion criteria: (1) Participants with other significant neurodegenerative diseases; (2) Interventions that were primarily based on music, dance, or drama, or multimodal expressive arts in which visual art was not a main component; (3) Non-RCTs, such as reviews, qualitative studies, case reports, and observational studies; (4) Studies for which the full text was unavailable or for which data could not be extracted.

### 2.4 Study selection and data extraction

Two researchers (YJ and YZ) independently screened the titles/abstracts and extracted data from the studies. Any discrepancies in study selection or data extraction were discussed and resolved by involving a third researcher until consensus was achieved. The collected data elements included each study’s basic characteristics, participant details, intervention protocols (frequency and duration), control conditions, outcome measures, and participant adherence information.

In accordance with the guidance in the Cochrane Handbook (32), when a study included multiple control arms, we prioritized a no-intervention control group, followed by an active control group that did not include visual art therapy.

Outcome measures: For global cognitive function, we prioritized the Montreal Cognitive Assessment (MoCA); if MoCA was not available, we used the Mini-Mental State Examination (MMSE) or other standardized cognitive assessment instruments; For memory function, the preferred measures were the Auditory Verbal Learning Test – Delayed Recall (AVLT-DR) and Auditory Verbal Learning Test – Immediate Recall (AVLT-IR), followed by other standardized memory assessment tools; For language function, the preferred measure was Verbal Fluency Test (VFT), followed by the MMSE-Language or other standardized language function assessment tools; For depressive symptoms, the preferred measure was the Geriatric Depression Scale (GDS) or the Geriatric Depression Scale-Short Form (GDS-SF), followed by other standardized depression assessment tools; For activities of daily living, the preferred measure was ADL or Instrumental Activities of Daily Living scale (IADL), followed by other standardized tools for assessing daily living activities.

### 2.5 Assessment of study quality

This study used the Cochrane RoB 2.0 tool (Risk of Bias 2) to assess the risk of bias in the included RCTs, focusing on the outcome level. The overall risk of bias was judged in line with the RoB 2.0 guidance (33). Specifically: (1) when all domains were assessed as low risk, the study was classified as having an overall low risk of bias; (2) when at least one domain was judged as having some concerns but none were judged high risk, the overall risk of bias was rated as some concerns; (3) when any domain was judged high risk, or when multiple domains raised some concerns that, in combination, substantially reduced confidence in the results for that outcome, the overall risk of bias was rated as high. Two reviewers independently assessed the studies and resolved disagreements through discussion, with a third-party adjudicator involved if necessary.

### 2.6 Certainty of evidence

The certainty of evidence for each outcome was assessed using the Grading of Recommendations Assessment, Development and Evaluation (GRADE) approach, and Summary of Findings (SoF) tables were generated using GRADEpro GDT. Because all included studies were RCTs, the certainty of evidence was initially rated as high and was downgraded as appropriate across five domains: risk of bias, inconsistency, indirectness, imprecision, and publication bias (34). The SoF tables present pooled effect estimates at post-intervention. Global cognition was specified as the primary outcome, and secondary outcomes included memory function, language function, depressive symptoms, and activities of daily living. Two reviewers (YJ and YZ) independently rated the certainty of evidence, and disagreements were resolved through discussion until consensus was reached.

### 2.7 Statistical analysis

All outcomes in this study were continuous variables. A fixed-effect model is appropriate when the true effect is assumed to be common across studies, whereas a random-effects model allows the true effects to vary between studies and incorporates between-study variance (35, 36). Given the heterogeneity in VAT research with respect to intervention protocols, comparator conditions, and outcome measurements, a random-effects model was applied to estimate pooled intervention effects across studies. Because only a small number of trials reported outcomes at multiple time points, to enhance consistency and comparability across studies, we pooled data at the post-treatment assessment at the end of the intervention. For each study, effect sizes were preferentially calculated using pre- to post-intervention change scores. When the standard deviation of the change score for a given outcome was not reported, it was imputed from baseline and post-intervention standard deviations by assuming a within-group pre–post correlation coefficient (*r*) (primary analyses: *r* = 0.50; sensitivity analyses: *r* = 0.25 and *r* = 0.75; detailed methods and results are provided in Supplementary Material 3). MD was used when outcomes were measured on the same scale with the same units; otherwise, SMD was used when different scales and/or units were applied. Between-study heterogeneity was assessed using Cochran’s *Q* test and the *I*^2^ statistic. Statistically significant heterogeneity was considered present when *I*^2^ > 50% or when the *Q* test *p*-value < 0.10. Where substantial heterogeneity was detected, subgroup and/or sensitivity analyses were conducted to explore potential sources. All statistical analyses were performed using RevMan 5.3. Pooled effects were tested using two-sided significance tests, and *p* < 0.05 was considered statistically significant.

## 3 Results

### 3.1 Study selection

**Figure 1:**
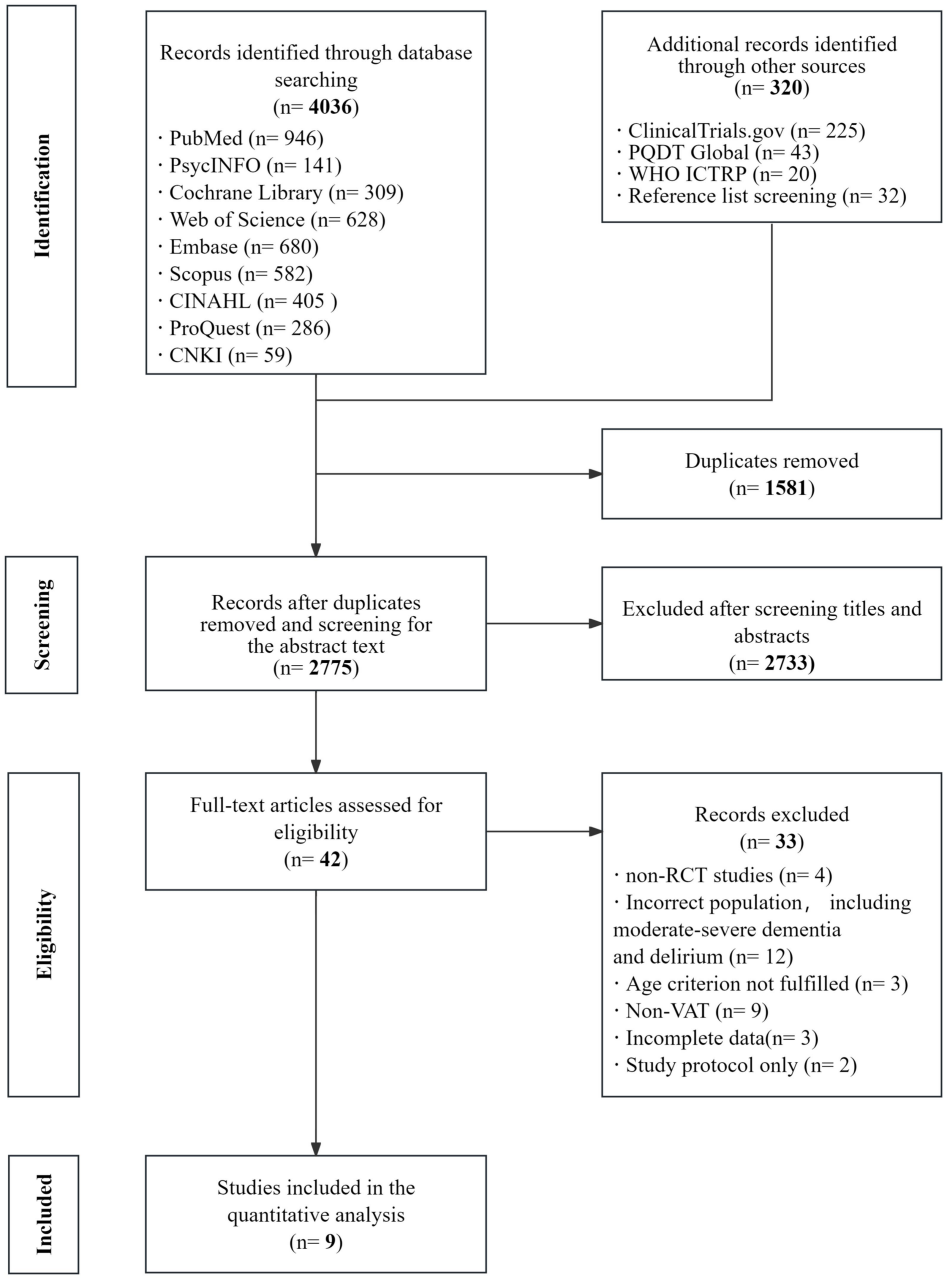
Literature selection process for systematic review.

The study selection process and results are presented in Figure 1. Database searches identified 4,036 potentially relevant records. An additional 288 records were retrieved from other sources to capture gray literature, and 32 records were identified through reference list screening. After removing 1,581 duplicate records, 2,775 records remained for title/abstract screening, of which 2,733 were excluded for not meeting the eligibility criteria. Full texts of the remaining 42 articles were assessed, and 33 studies were further excluded, primarily because they were not randomized controlled trials, involved participants with moderate-to-severe dementia or delirium, did not meet the age criterion, evaluated interventions that were not VAT, reported incomplete outcome data, or were study protocols only. Ultimately, nine studies were included in the systematic review and quantitative synthesis (13, 14, 21, 37–42).

### 3.2 Characteristics of included studies

The main characteristics of the included trials are summarized in Table 2. Five studies were conducted in China, two in Tanzania, and the remaining two were conducted in South Korea and Singapore, respectively. Overall, the nine studies included 595 participants, with 299 allocated to the VAT group and 296 to the control group. Across included studies, the mean intervention duration was approximately 14 weeks, and the mean total contact time per study was approximately 26 h.

**Table 2:** Study characteristics.

Source	Country	Diagnostic tool	Group size, female (%)		Age, mean (SD)		Intervention group			Control condition	Outcomes	Dropout (n)	
			IG	CG	IG	CG	Method	Length	Total contact time			IG	CG
Huang et al. (2025) (37)	China	Petersen criteria	40 (77.5%)	40 (65%)	87.5	85	VAT (painting, material exploration) 2 sessions/week, 60 min/session	14 wk	28 h	Usual care	MoCA; AVLT-IR; VFT	5	7
Kwok et al. (2011) (42)	China	MMSE	14 (64.3%)	17 (88.2%)	85.79 (4.93)	85.76 (6.93)	Calligraphy therapy. 5 sessions/week, 30 min/session	8 wk	20 h	No intervention	MMSE; AVLT-DR; MMSE-Language	0	0
Lee et al. (2022) (38)	South Korea	MoCA; MMSE	20 (50.0%)	19 (57.9%)	75.45 (6.38)	76.42 (5.25)	VAT (created artworks using food materials). 3 sessions/week, 120 min/session	4 wk	24 h	Usual care	MoCA; SGDS-K; K-IADL	2	4
Lin et al. (2022) (39)	China	Not specified	45 (66.7%)	45 (55.6%)	70.98 (6.45)	69.22 (6.91)	VAT (painting, collage, drawing, clay modeling, and storytelling). 1 session/week, 90 min/session	24 wk	36 h	Wait-list	MoCA; AVLT-DR; VFT; GDS; ADL	3	6
Luo et al. (2023) (40)	China	Petersen criteria	38 (74%)	35 (57%)	71.5	71	VAT (visual art creation, storytelling). 2 sessions/week, 60 min/session	12 wk	24 h	Health education	MoCA; MMSE; AVLT-DR; AVLT-IR; VFT; ADL	1	3
Mahendran et al. (2018)* (13)	Singapore	Petersen criteria	22 (81.8%)	22 (81.8%)	71.1 (4.8)	70.6 (5.8)	VAT (visual art creation). 1 session/week, 60 min/session	36 wk	36 h	Usual care	MoCA; AVLT-DR; GDS-SF	3	4
Masika et al. (2020) (21)	Tanzania	DSM-5	21 (85.7%)	18 (88.9%)	73.4 (7.3)	72 (7.1)	VAT based on Zentangle methods. 2 sessions/week, 120 min/session	6 wk	24 h	Health education	MoCA-5-min; GDS-SF	3	0
Masika et al. (2022) (41)	Tanzania	DSM-5	62 (82.3%)	65 (73.8%)	73.6 (7.6)	74.1 (8.3)	VAT based on Zentangle methods. 2 sessions/week, 120 min/session	6 wk	24 h	Health education	MoCA-5-min; GDS-SF; IADL	8	9
Zhao et al. (2018) (14)	China	DSM-IV	48 (52.1%)	45 (51.1%)	70.6	69.5	VAT (viewing pictures, storytelling, drawing). 1 session/week, 60 min/session	16 wk	16 h	Cognitive training	MoCA; AVLT-DR; AVLT-IR; VFT; ADL	3	2

MoCA, Montreal Cognitive Assessment; MMSE, Mini-Mental State Examination; MMSE-Language, Language subscore of the Mini-Mental State Examination; AVLT-DR, Auditory Verbal Learning Test–Delayed Recall; VFT, Verbal Fluency Test; GDS, Geriatric Depression Scale; GDS-SF, Short-form Geriatric Depression Scale (15-item); SGDS-K, Korean version of the Short Geriatric Depression Scale; ADL, Activities of Daily Living; K-IADL, Korean Instrumental Activities of Daily Living scale; MoCA-5-min, Montreal Cognitive Assessment–5-Minute Protocol; IG, intervention group; CG, control group.

^*^This study included two active intervention arms, namely a VAT arm and a music nostalgia therapy arm. Because this review specifically focused on VAT, only participants allocated to the VAT arm were included in the analysis.

### 3.3 Results of the risk of bias assessment

**Figure 2:**
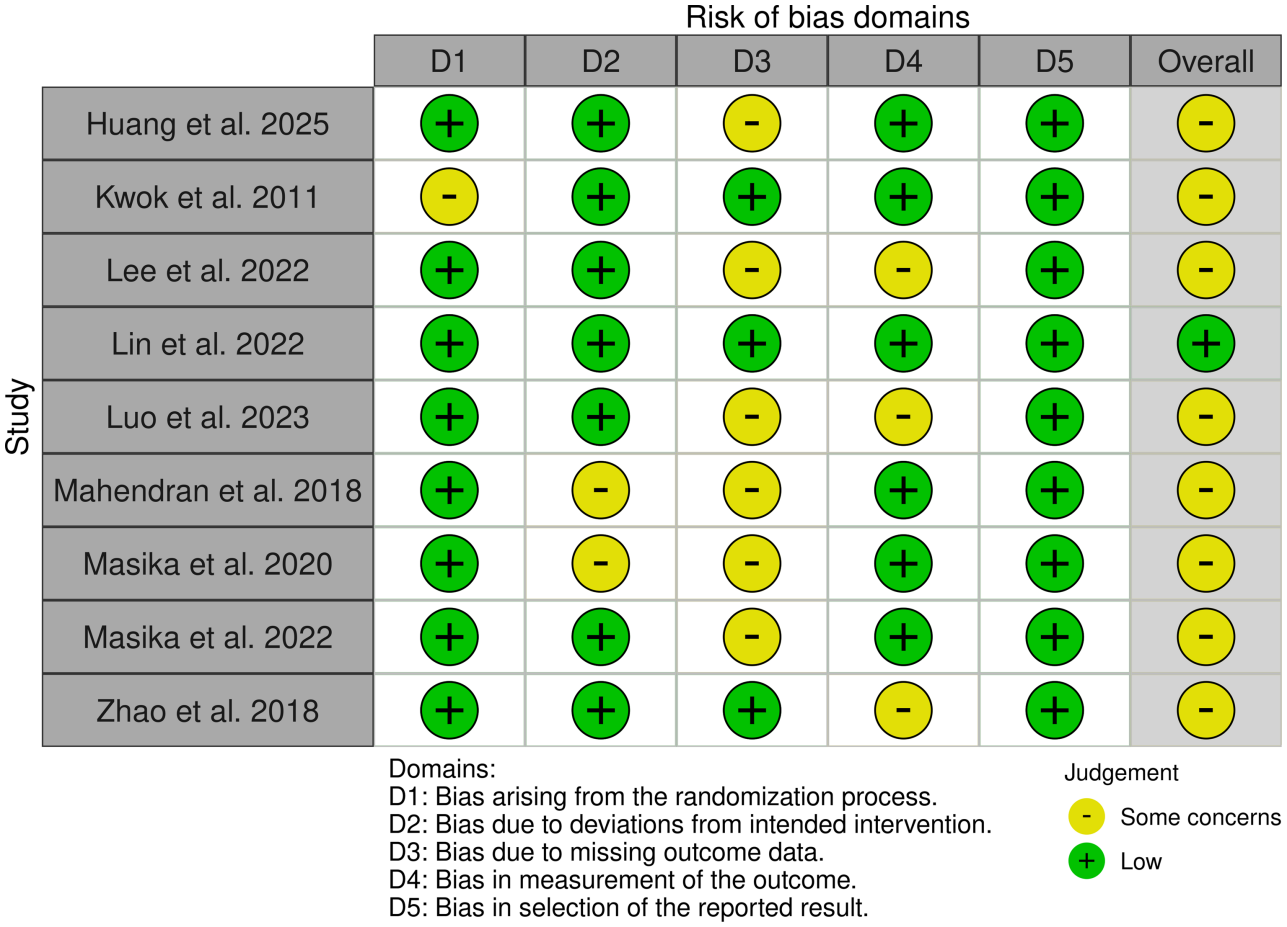
Risk of bias traffic-light plot.

The detailed risk-of-bias assessments are presented in Table 3 and Figure 2. Among the included studies, eight trials (13, 14, 21, 37, 38, 40–42) were rated as having some concerns, whereas one trial (39) was judged to be at low risk of bias. Specifically: (1) Regarding bias arising from the randomization process, most studies clearly reported sequence generation and allocation concealment; one study (42) did not sufficiently describe these details and was therefore rated as having some concerns. (2) With respect to bias arising from deviations from the intended interventions, most trials used active or attention-control conditions, and no evidence of systematic contamination between groups was observed. Two studies (13, 41) adopted an open-label design, and the control arms did not receive interventions matched in intensity to those of the intervention groups. These studies had potential expectancy effects or behavioral compensation, but the use of blinding/intention-to-treat (ITT) analysis partially mitigated the bias. (3) With respect to bias related to missing outcome data, 6 studies (13, 21, 37–39, 41) had moderate dropout or attrition rates during follow-up, with some imbalance between groups. These studies mostly used Generalized Estimating Equations (GEE)/mixed models to include incomplete data and reduce bias, but the precision of long-term effect estimates remained affected by uncertainty. Three studies (14, 40, 42) had complete or near-complete follow-up, with no systematic missing data patterns related to outcomes or groups, resulting in lower risk. (4) Regarding bias in outcome measurement, most studies implemented blinding for assessors, with primary outcomes being objective measures that were standardized and less susceptible to subjective influence. Even if participants were aware of their group assignment, the measurements remained relatively controlled. Three studies (14, 38, 39), while using blinded assessors for most objective measures, included some subjective outcomes that could be influenced by participants’ knowledge of their group assignment. Potential impacts on subjective outcomes should be interpreted with caution. (5) Regarding selective reporting bias, all studies reported primary and secondary outcomes according to predefined protocols and time points, with no selective reporting observed, even for outcomes with no significant differences.

**Table 3:** Results of bias assessment.

Study	Randomization process	Deviations from intended interventions	Missing outcome data	Measurement of outcome	Selection of reported result	Overall risk
Huang et al. (2025) (37)	Low risk	Low risk	Some concerns	Low risk	Low risk	Some concerns
Kwok et al. (2011) (42)	Some concerns	Low risk	Low risk	Low risk	Low risk	Some concerns
Lee et al. (2022) (38)	Low risk	Low risk	Some concerns	Some concerns	Low risk	Some concerns
Lin et al. (2022) (39)	Low risk	Low risk	Low risk	Low risk	Low risk	Low risk
Luo et al. (2023) (40)	Low risk	Low risk	Some concerns	Some concerns	Low risk	Some concerns
Mahendran et al. (2018) (13)	Low risk	Some concerns	Some concerns	Low risk	Low risk	Some concerns
Masika et al. (2020) (21)	Low risk	Some concerns	Some concerns	Low risk	Low risk	Some concerns
Masika et al. (2022) (41)	Low risk	Low risk	Some concerns	Low risk	Low risk	Some concerns
Zhao et al. (2018) (14)	Low risk	Low risk	Low risk	Some concerns	Low risk	Some concerns

Overall, the risk of bias across the included studies was predominantly some concerns, and the findings should be interpreted with caution.

### 3.4 Efficacy of VAT

#### 3.4.1 Global cognitive function

**Figure 3:**
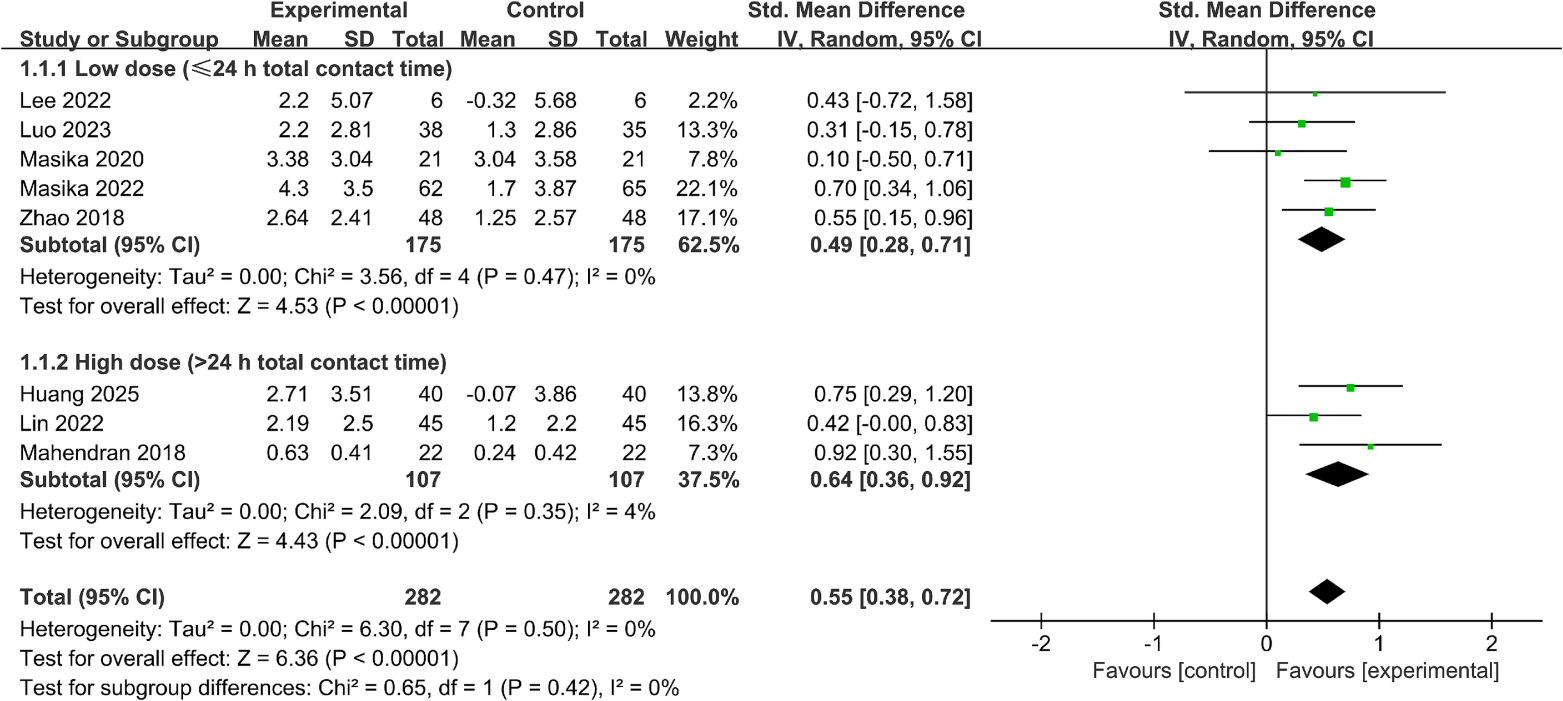
Impact of VAT on global cognitive performance (MoCA).

Eight studies (*n* = 564) reported outcomes on global cognitive function assessed using the MoCA (see Figure 3). Pooled results showed that, compared with controls, VAT improved global cognitive performance (SMD = 0.55, 95% CI 0.38 to 0.72, *p* < 0.05; *I*^2^ = 0%). In the dose subgroup analysis by total contact time (≤24 h vs. >24 h), both subgroups reached statistical significance: the lower-dose subgroup (≤24 h) had an SMD of 0.49 (95% CI 0.28 to 0.71; *I*^2^ = 0%), and the higher-dose subgroup (>24 h) had an SMD of 0.64 (95% CI 0.36 to 0.92; *I*^2^ = 4%). The test for subgroup differences was not statistically significant (*p* = 0.42), indicating that the current evidence is insufficient to support a clear dose–response gradient. When studies were subgrouped by comparator type (see Supplementary Material 3), the pooled effect size was SMD = 0.63 (95% CI 0.36 to 0.89, *p* < 0.05; *I*^2^ = 0%) in trials with passive controls, and SMD = 0.49 (95% CI 0.25 to 0.72, *p* < 0.05; *I*^2^ = 0%) in trials with active controls. The subgroup difference test showed no statistically significant difference between comparator types (*p* = 0.44), suggesting that comparator intensity may not have materially influenced the overall effect estimate.

**Figure 4:**
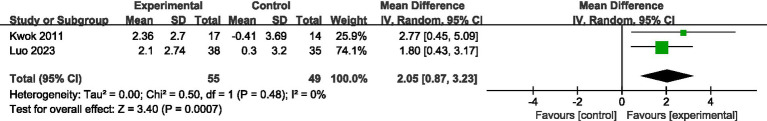
Impact of VAT on global cognitive performance (MMSE).

Given the small number of MMSE-based studies and the possibility that the MMSE is less sensitive than the MoCA to subtle cognitive changes at the MCI stage, MMSE results were synthesized separately as supplementary evidence and should be interpreted with caution. Two studies (*n* = 104) were included (see Figure 4). The pooled analysis suggested that VAT may improve MMSE scores (MD = 2.05, 95% CI 0.87 to 3.23, *p* < 0.05; *I*^2^ = 0%).

In sensitivity analyses assuming different pre–post correlation coefficients (*r* = 0.25 and *r* = 0.75), the direction and statistical significance of the pooled effect for the primary outcome remained consistent, indicating that the findings were generally robust to the choice of *r* (see Supplementary Material 3). Overall, based on the available evidence, VAT may improve cognitive function in older adults with MCI.

#### 3.4.2 Memory function

**Figure 5:**
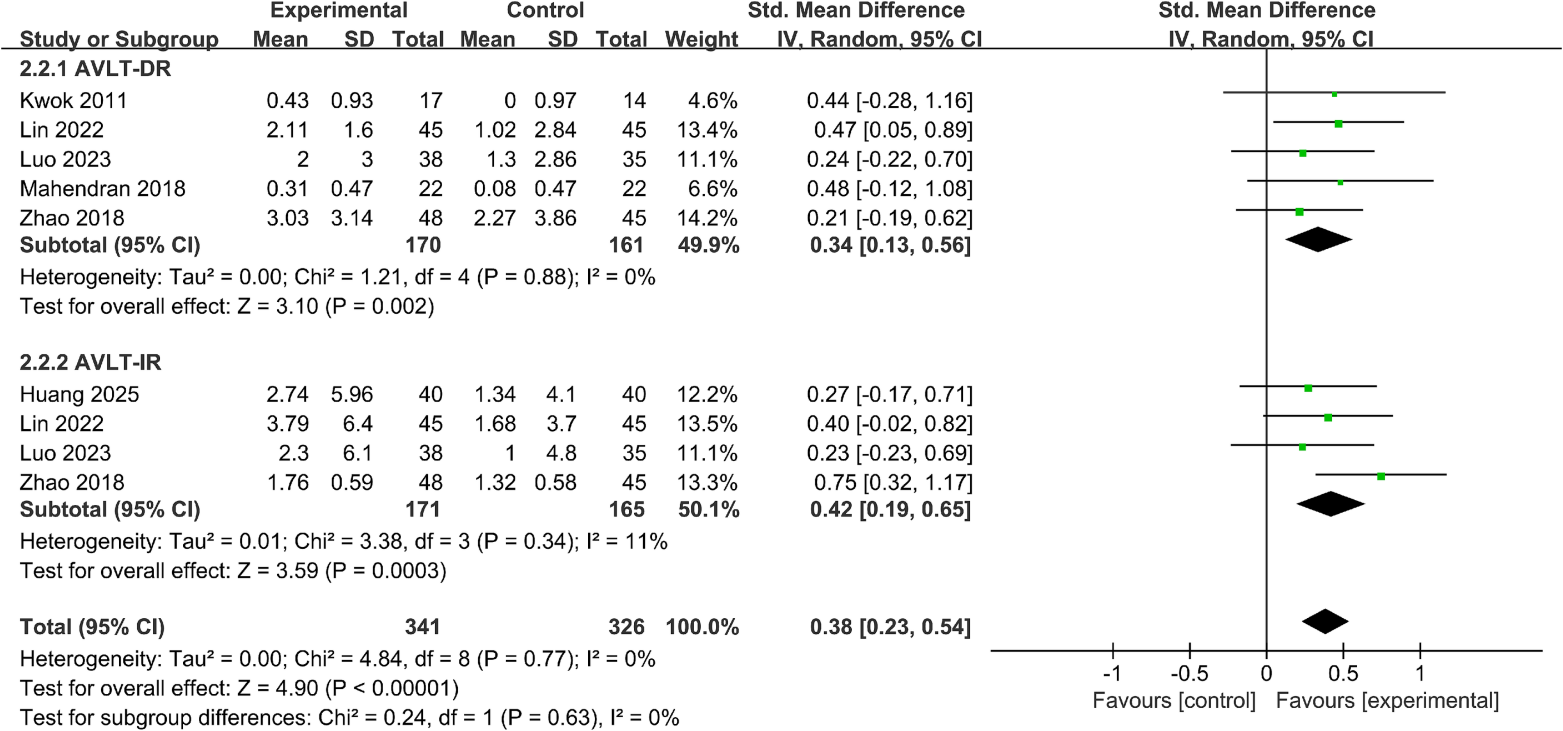
Impact of VAT on memory function.

Five studies (*n* = 331) reported outcomes on long-term memory (see Figure 5). Meta-analysis showed that, compared with controls, VAT significantly improved long-term memory performance in participants with MCI (SMD = 0.34, 95% CI 0.13 to 0.56, *p* < 0.05; *I*^2^ = 0%).

Four studies (*n* = 336) reported outcomes on short-term memory (see Figure 5). The pooled results indicated that, compared with control conditions, VAT significantly improved short-term memory in participants with MCI (SMD = 0.42, 95% CI 0.19 to 0.65, *p* < 0.05; *I*^2^ = 11%).

In sensitivity analyses assuming different pre–post correlation coefficients, the direction and statistical significance of the pooled effects for these outcomes remained consistent, suggesting that the findings were robust to the choice of the correlation coefficient (see Supplementary Material 3).

#### 3.4.3 Language function

**Figure 6:**
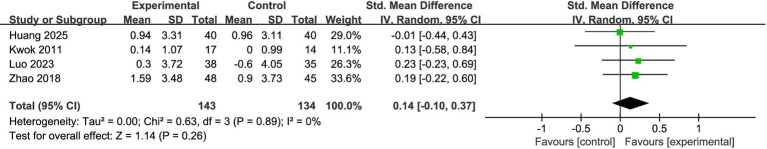
Impact of VAT on language function.

Four studies (*n* = 277) reported outcomes on language function (see Figure 6). Meta-analysis showed that, compared with controls, VAT did not produce a statistically significant improvement in language function among participants with MCI (SMD = 0.14, 95% CI −0.10 to 0.37, *p* > 0.05; *I*^2^ = 0%). These findings suggest that, based on the current evidence, the benefit of VAT on language function in individuals with MCI remains uncertain. In sensitivity analyses assuming different pre–post correlation coefficients, the direction and statistical significance of the pooled effect for this outcome remained consistent, indicating robustness to the choice of the correlation coefficient (see Supplementary Material 3).

#### 3.4.4 Depressive symptoms

**Figure 7:**
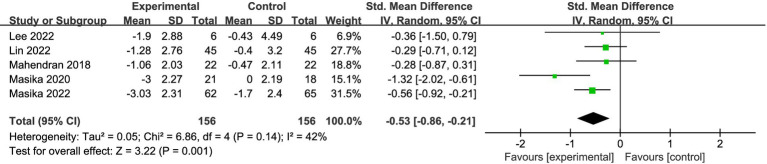
Impact of VAT on depressive symptoms.

Five studies (*n* = 312) reported outcomes on depressive symptoms (see Figure 7). The random-effects meta-analysis showed that, compared with controls, VAT significantly reduced depressive symptoms in participants with MCI (SMD = −0.53, 95% CI −0.86 to −0.21, *p* < 0.05). There was moderate between-study heterogeneity (*I*^2^ = 42%, *p* = 0.14). Given the small number of included studies for this outcome, subgroup analyses or meta-regression were considered underpowered and potentially unstable; therefore, we conducted a leave-one-out sensitivity analysis to evaluate the influence of individual studies on the pooled effect and heterogeneity.

The sensitivity analysis indicated that the study by Masika et al. (21) contributed substantially to heterogeneity. Specifically, this trial showed a marked post-randomization baseline imbalance in depressive symptom severity (GDS-SF: 4.67 ± 2.337 vs. 3.44 ± 1.867), and its effect size deviated notably from those of the other studies. After excluding this study, heterogeneity was eliminated (*I*^2^ = 0%, *p* = 0.75), while the pooled effect remained statistically significant (SMD = −0.42, 95% CI −0.66 to −0.18, *p* < 0.05).

In sensitivity analyses assuming different pre–post correlation coefficients, the direction and statistical significance of the pooled effect for this outcome remained consistent (see Supplementary Material 3). Overall, the available evidence suggests that VAT may help alleviate depressive symptoms in older adults with MCI.

#### 3.4.5 Activities of daily living

**Figure 8:**
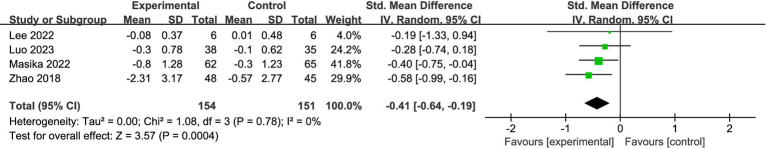
Impact of VAT on activities of daily living.

Four studies (*n* = 305) reported outcomes on activities of daily living (see Figure 8). Meta-analysis showed that, compared with controls, VAT significantly improved activities of daily living among participants with MCI (SMD = −0.41, 95% CI −0.64 to −0.19, *p* < 0.05; *I*^2^ = 0%). In sensitivity analyses assuming different pre–post correlation coefficients, the direction and statistical significance of the pooled effect for this outcome remained consistent, indicating robustness to the choice of the correlation coefficient (see Supplementary Material 3).

#### 3.4.6 Assessment of publication bias

**Figure 9:**
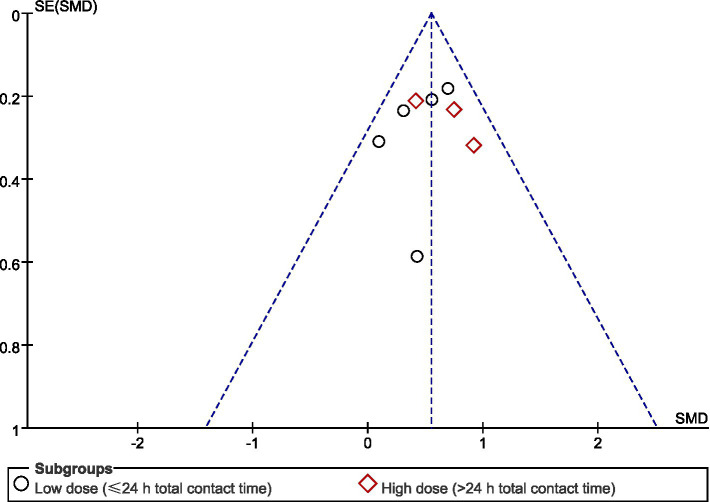
Funnel plot for the assessment of publication bias.

To assess potential publication bias for the primary outcome, a funnel plot was constructed (see Figure 9). The funnel plot did not suggest marked asymmetry, and Egger’s regression test also provided no evidence of funnel plot asymmetry (intercept = −0.58, *p* = 0.67). However, it should be emphasized that, because the number of included studies was small (*n* = 8), visual inspection of funnel plot symmetry and Egger’s test are underpowered and may yield unstable results in this context. Therefore, these findings should be interpreted with caution and do not rule out the possibility of publication bias or small-study effects.

#### 3.4.7 Summary of findings and certainty of evidence

The certainty of evidence was assessed using GRADE approach and is presented in the SoF table (see Table 4). The certainty of evidence for global cognition was rated as moderate, whereas the certainty for memory domains, depressive symptoms, activities of daily living, and language outcomes was rated as low, mainly due to concerns regarding risk of bias and imprecision.

**Table 4:** Summary of findings.

Outcomes	Certainty assessment						No. of patients		Effect (95% CI)	Certainty
	No. of studies	Risk of bias	Inconsistency	Indirectness	Imprecision	Publication bias	Experimental	Control		
Global cognitive function (MoCA)	8	Serious^a^	Not serious	Not serious	Not serious	No clear publication bias detected, but limited by few studies	282	282	SMD **0.55 higher** (0.38 higher to 0.72 higher)	⨁⨁⨁◯ Moderate^a^
Memory function (Long-term memory)	5	Serious^a^	Not serious	Not serious	Serious^b^	Undetected	170	161	SMD **0.34 higher** (0.13 higher to 0.56 higher)	⨁⨁◯◯ Low^a, b^
Memory function (Short-term memory)	4	Serious^a^	Not serious	Not serious	Serious^b^	Undetected	171	165	SMD **0.42 higher** (0.19 higher to 0.65 higher)	⨁⨁◯◯ Low^a, b^
Language function	4	serious^a^	not serious	not serious	serious^b, c^	undetected	143	134	SMD **0.14 higher** (−0.10 higher to 0.37 higher)	⨁⨁◯◯ Low^a, b, c^
Depressive symptoms	5	Serious^a^	Not serious	Not serious	Serious^b^	Undetected	156	156	SMD **0.53 lower** (0.86 lower to 0.21 lower)	⨁⨁◯◯ Low^a, b^
Activities of daily living	4	Serious^a^	Not serious	Not serious	Serious^b^	Undetected	154	151	SMD **0.41 lower** (0.64 lower to 0.19 lower)	⨁⨁◯◯ Low^a, b^

^a^The RoB 2 assessment of the included RCTs indicated that most studies had “some concerns”; ^b^The pooled sample size was small; ^c^Confidence interval crossing the clinical decision threshold. Boldface in this table reflects the default formatting generated by GRADEpro GDT to highlight the summary effect estimates and the direction of effect for the intervention group relative to the control group on the analyzed scale; it does not indicate additional statistical significance or special emphasis by the authors.

## 4 Discussion

### 4.1 Discussion of global cognitive function

This systematic review suggests that VAT may improve global cognitive function in older adults with MCI. This result aligns with prior evidence. For example, the systematic reviews conducted by Fong et al. (23) and Yue et al. (25) reported that most art-based interventions were associated with improvements in global cognitive performance among older individuals with MCI. Studies specifically on VAT have also reported similar conclusions. In a review by Chiang et al. (26), 5 out of 7 studies observed improvements in cognitive scores, with two studies maintaining cognitive gains at 6–9 months follow-up. The systematic review by Masika et al. (21) reported that VAT produces notable cognitive benefits in older adults, with particularly pronounced effects in those with MCI. Taken together, the available evidence indicates that VAT contributes to improving global cognitive function in this population.

The observed improvement in global cognitive function following VAT may be attributable to its integrated cognitive stimulation effects. Based on the existing evidence, we propose the following hypothesis-driven explanations. On the one hand, engaging in artistic creation—such as painting or sculpting—simultaneously taps into multiple cognitive domains, including memory, attention, executive functioning, and visuospatial processing. During the creative process, multiple sensory and cognitive pathways in the brain are simultaneously activated, promoting neural connectivity and plasticity (26, 43, 44). This process is similar to complex cognitive training, which helps improve the overall function of the brain. On the other hand, many VAT interventions are conducted in group settings, which involve discussion and social interaction. The research by Huang et al. (37) pointed out that art interventions with significant cognitive improvement often incorporate social elements, such as group discussions on creative inspiration and emotional experiences. Social interaction itself benefits cognitive health, reduces feelings of loneliness, and provides cognitive stimulation, making it one of the potential mechanisms for cognitive improvement (45). Additionally, the pleasure and sense of accomplishment derived from art creation may reduce psychological stress and depression levels, and this emotional improvement can indirectly enhance cognitive performance (46, 47). Overall, VAT may enhance global cognitive function in individuals with MCI through multiple pathways, including direct cognitive practice as well as indirect effects via social engagement and emotion regulation.

## 5 Discussion of memory

This review stratified memory outcomes by instrument into long-term memory (delayed recall) and short-term memory (immediate recall). The meta-analytic findings indicate that VAT confers benefits on both domains in older adults with MCI. This aligns with prior evidence: in the study by Fong et al. (23), more than half of included interventions reported improvements in memory—particularly in learning and recall—among older adults with MCI; VAT-focused evidence is consistent as well. For example, after a 12-session Zentangle drawing program, Masika et al. (41) observed significantly higher scores across memory subscales in the intervention group versus controls, with gains maintained at 3- and 9-month follow-up. In addition, an MRI-based randomized controlled trial (48) found that participants in the VAT arm exhibited increased cortical thickness in the middle frontal gyrus, accompanied by functional improvements, thereby providing neuroimaging evidence supporting VAT-related memory enhancement. Not all trials found between-group differences, however: in Zhao et al. (14), memory improved within the VAT group but did not differ significantly from controls, likely because the control arm received cognitive training of comparable frequency and intensity. Variability in memory outcomes across studies may therefore reflect differences in intervention content or emphasis and the nature of control conditions. Synthesizing these results with prior evidence, our study further supports VAT as an effective strategy for memory enhancement in this population.

The association between VAT and improvements in memory may involve multiple mechanisms. Based on the existing evidence, we propose the following hypothesis-driven explanations. First, the act of art-making inherently recruits memory processes: participants often draw on autobiographical experiences for inspiration and must remember procedural steps and compositional plans, thereby exercising retrieval and storage capacities (46, 49). Second, neurobiological work suggests that art activities can stimulate hippocampal circuits and strengthen large-scale network connectivity; sustained practice may promote synaptic plasticity and consolidate memory traces (50, 51). Third, emotionally salient creation fosters deeper encoding; affectively meaningful material is more likely to undergo elaborative processing and yield durable memories (52). Finally, VAT tasks place continuous demands on working memory—holding layouts in mind, sequencing strokes or materials, and manipulating intermediate representations—thereby training immediate storage and mental operations; repeated practice may incrementally strengthen working memory capacity (39). Overall, VAT may enhance memory function in older adults with MCI by providing enriched cognitive stimulation and affective experiences; however, these hypotheses require further confirmation in future studies incorporating mechanistic measures and/or mediation analyses.

### 5.1 Discussion of language function

The meta-analysis did not show a statistically significant effect of VAT on post-intervention language function in older adults with MCI. This result appears to differ from some prior reports. For instance, Johnson et al. (52) observed within-group improvements on VFT and Boston Naming Test (BNT) following an art-based program in this population. Similarly, in a RCT by Luo et al. (40), a 12-week course of art therapy produced significantly greater gains on VFT scores in MCI participants compared with the control group. Likewise, the systematic review by Hu et al. (22) noted that studies incorporating language measures frequently reported improvements. These observations suggest that under certain conditions, art-based interventions may enhance language abilities in older adults. By contrast, our findings did not support an overall improvement in language function with VAT among older adults with MCI. Three factors may account for this: first, VAT is primarily visual and hands-on. Although some programs include discussion and interaction, the core modality is non-linguistic. In many drawing or craft activities, expression occurs mainly through visual–motor channels, so language output is not the training focus, and direct stimulation of language processes is limited. By comparison, drama or music may engage language abilities more directly. Second, relatively few studies treated language function as an outcome and sample sizes were small, resulting in limited statistical power to detect effects in the pooled analysis. Third, language function in early MCI is often relatively preserved (most MCI presentations feature deficits in memory or executive function and verbal fluency is often less affected), leaving little room for improvement from near-normal baselines. These factors together may explain why we did not observe significant changes in language outcomes. Future studies are therefore needed to further clarify the impact of VAT on language function in this population.

### 5.2 Discussion of depressive symptoms

The meta-analysis suggests that VAT significantly reduces depressive symptoms in older adults with MCI. This is broadly in line with the existing body of evidence. In the systematic review by Xu et al. (24), 7 of 9 included studies reported significant reductions in depressive symptoms following art-based interventions. Similarly, Chiang et al. (26) found that, among six studies assessing mental health outcomes, four reported beneficial effects of VAT on participants’ psychological well-being. Consistent results have also been observed in dementia populations: VAT has been shown to reduce depressive symptoms and hopelessness and to elicit positive affective experiences such as satisfaction and calm (22). However, not all studies observed clear mood benefits. In a study using dance as the sole modality (53), scores for depression and anxiety improved in the intervention arm but did not reach statistical significance. The authors argued that single-modality interventions may have a limited impact and recommended integrating multiple art forms and employing more sensitive assessment tools. This pattern suggests that emotional outcomes may vary by modality; in relative terms, more comprehensive programs—or longer intervention periods—may be more likely to yield measurable improvements (24). Overall, our conclusions accord with the mainstream view that VAT, as a non-pharmacological approach, has the potential to relieve depressive symptoms and meaningfully promote mental health in this population.

The association between VAT and improvements in depressive symptoms may be explained through two potential pathways: psychological and social mechanisms. Psychological level: creative activities such as painting and sculpture provide a nonverbal channel for emotional expression, allowing negative affect to be released—a process akin to “catharsis” and “self-healing” (13, 26). Art making also engenders pleasure and a sense of accomplishment (46, 47). When individuals with MCI engage deeply in drawing or crafts, they may experience a state of flow, which can temporarily reduce anxiety about cognitive decline and lower stress levels (41). Social level: the social components of group-based VAT are equally important. Depressive symptoms are often linked to loneliness and social isolation, particularly among older adults with cognitive impairment (13, 54, 55). Group art classes create opportunities for interaction; collaborative creation and sharing of works help establish supportive social ties—an influential mechanism behind emotional improvement (42, 45). Notably, implementation details matter: granting participants autonomy, allowing choice of preferred materials and themes, and encouraging active self-expression may enhance therapeutic benefits (24). Overall, VAT may alleviate depressive symptoms in older adults with MCI by providing a platform for emotional expression, fostering pleasurable experiences, and enhancing social support, thereby benefiting psychological well-being through multiple dimensions.

### 5.3 Discussion of activities of daily living

The meta-analysis indicate that VAT is associated with improved activities of daily living in older adults with MCI. This finding aligns with part of the prior literature. For example, the systematic review by Masika et al. (21) reported better daily functioning in VAT groups than in controls, especially in creative art therapy programs. Chiang et al. (26) recently reported in a review that art-based interventions may help preserve or enhance functional ability in individuals with MCI. These convergent findings may reflect VAT’s combined effects on cognition and mood. Engaging in visual art tasks can strengthen memory and executive function (supporting planning and task completion), while the enjoyable creative experience may reduce depressive symptoms, boost engagement and self-confidence, thereby translating into better ADL performance.

Evidence regarding activities of daily living outcomes was not entirely consistent, indicating that the functional benefits of VAT may differ across studies. In particular, neither the follow-up trial by Masika et al. (41) nor the study conducted by Luo et al. (40) showed statistically significant ADL differences between participants receiving VAT and those in the control groups. Several factors may explain these discrepancies: (1) Near-normal baseline ADL in MCI participants leaves limited room for improvement; (2) Heterogeneity of protocols and measures (e.g., differences in VAT duration, frequency, content, and varying sensitivity of ADL instruments); (3) Small samples and short follow-up reduce power to detect longer-term functional change. Overall, our findings indicate that VAT may help support daily functioning in older adults with MCI, but its effectiveness is likely influenced by multiple contextual and methodological factors. To substantiate these effects and better understand the underlying mechanisms, larger, rigorously designed trials with longer follow-up periods and more refined outcome assessments are still needed.

### 5.4 Public health implications

From a public health perspective, VAT—as a non-pharmacological intervention—appears to be feasible and scalable in community and primary care settings and has the potential to be incorporated into routine care pathways. First, in terms of feasibility, VAT typically relies on visual art media with low technical requirements; material costs are relatively controllable, and delivery is often organized in structured group formats (29, 56, 57). In communities with basic space and organizational capacity, VAT delivered by therapists or standardized trained facilitators, and operated under professional supervision, may help provide accessible services under resource-constrained conditions; Second, regarding scalability, group-based delivery can increase coverage efficiency per unit of workforce. The use of brief and replicable procedures, together with standardized themes and material checklists, may facilitate dissemination across different community sites and improve intervention consistency (37, 58); In terms of implementation, primary care and community services may embed VAT within a continuous “screening–referral–intervention–follow-up” management chain: after identifying individuals with MCI, referrals can be made to community group-based VAT programs (59), with process monitoring of attendance, adherence, and adverse events, and coordination with other supportive services when needed (e.g., psychological support, cognitive training, or caregiver guidance) (60). It should be emphasized that existing studies vary in intervention dose, provider qualifications, and comparator conditions, and evidence on long-term follow-up and cost-effectiveness remains limited. Therefore, the above considerations should be viewed as practice implications derived from the current evidence base. Future research is warranted to further evaluate sustainability, accessibility, and implementation effectiveness in real-world settings.

### 5.5 Study limitations and directions for future research

This systematic review has several limitations. First, the overall sample sizes of the included studies were small, and the evidence base was largely derived from Asian settings; studies conducted in other cultural contexts and healthcare systems remain limited, which may restrict the generalizability of the findings. Second, due to the nature of art therapy interventions, blinding of participants and intervention providers is often difficult, which may introduce placebo or expectancy effects and increase the risk of bias. Third, outcomes in the present review were primarily based on rating-scale scores, with limited reporting of objective measures, thereby reducing objectivity and limiting the testability of mechanistic explanations. Fourth, differences across studies in MCI diagnostic criteria, comparator conditions, and the form and intensity of interventions may have introduced heterogeneity that could influence the pooled effect estimates. Fifth, although subgroup and sensitivity analyses were conducted to explore potential sources of heterogeneity, the small number of available studies and inconsistent reporting limited the feasibility of stable meta-regression or more granular stratified comparisons; thus, the ability to explain heterogeneity remained constrained. Finally, potential publication bias and language bias should be interpreted cautiously. Only Chinese- and English-language studies were included. Although gray literature searches and supplementary searches were undertaken, the number of included studies remained limited, and the reliability of funnel plot inspection and Egger’s test was restricted under these conditions; therefore, publication and language bias cannot be ruled out. Taken together, these considerations suggest that the results should be viewed as an evidence-based synthesis of the current literature and interpreted with caution.

For future research, several recommendations are proposed. First, larger-sample, multicenter RCTs across diverse cultural contexts are needed. Building on prospective protocol registration, such trials should strengthen randomization procedures and implement feasible blinding-related safeguards where possible, and report intervention details comprehensively in accordance with the Consolidated Standards of Reporting Trials (CONSORT) and the Template for Intervention Description and Replication (TIDieR) guidelines (e.g., session procedures, themes, material lists, provider training/supervision, and process fidelity) to enhance reproducibility and cross-study comparability. Second, regarding outcome selection, core clinical scales should be retained while incorporating more objective measures, such as task-based ADL/IADL assessments, computerized cognitive tests, and—when feasible—neurophysiological or neuroimaging markers and other biomarkers. Standardized reporting of adverse events, attendance, adherence, and other process indicators is also recommended to support safety evaluation and implementation-effectiveness assessment. Third, for comparator design, the use of a pre-specified active-control versus passive-control framework or more consistent comparator protocols is recommended; analysis plans should predefine stratified analyses by comparator intensity and cognitive instruments to reduce confounding arising from systematic between-study differences. Fourth, to clarify dose–response relationships and the durability of effects, study designs should more systematically manipulate and report intervention frequency, session duration, and total contact time, and include sufficiently long follow-up (e.g., ≥12 months) to assess long-term and maintenance effects. Where sample size permits, meta-regression or dose–response analyses based on individual participant data (IPD) may help identify active components and optimal parameter combinations. Fifth, given the public health potential for community implementation, pragmatic implementation studies and cost-effectiveness evaluations in real-world settings are needed to provide actionable evidence for integrating VAT into continuous community/primary-care management pathways.

## 6 Conclusion

This systematic review and meta-analysis suggest that VAT may improve global cognitive function in older adults with MCI, with moderate certainty of evidence for this primary outcome. For other secondary outcomes, including memory function, depressive symptoms, and activities of daily living, the certainty of evidence was rated as low, indicating possible benefits but substantial uncertainty. For language function, the certainty of evidence was also low, and the effect size was small with confidence intervals compatible with no meaningful benefit; therefore, no definitive conclusion can be drawn at present. Overall, VAT may be considered a potentially valuable adjunctive non-pharmacological intervention for older adults with MCI, but conclusions beyond global cognition should be interpreted with caution. Larger, more rigorously designed RCTs with more standardized outcome measurement and longer follow-up are needed to confirm these findings and to improve the certainty and generalizability of the evidence.

## Data Availability

The original contributions presented in the study are included in the article/Supplementary material, further inquiries can be directed to the corresponding author.
