# Supplementary material for: The effects of visual art therapy in older adults with mild cognitive impairment: a systematic review and meta-analysis
Source: Front Public Health. 2026 Mar 23;14:1765620. doi: 10.3389/fpubh.2026.1765620 (PMC13051655; doi:10.3389/fpubh.2026.1765620)
Supplement: Supplementary file 2 [file Data_Sheet_2.pdf]

Search strategies

1. **PubMed**

("art therapy" OR "visual art therapy" OR "visual art" OR "visual aesthetic" OR "art intervention" OR "creative art" OR "art psychotherapy" OR "painting" OR "drawing")
AND
("cognitive dysfunction" OR "cognitive impairment" OR "mild cognitive impairment" OR "mild cognitive decline" OR "MCI" OR "subjective cognitive complaint" OR "subjective memory impairment")
AND
("elderly" OR "older adults" OR "aged" OR "geriatric")

2. **PsycINFO**

("art therapy" OR "visual art therapy" OR "visual art" OR "visual aesthetic" OR "art intervention" OR "creative art" OR "art psychotherapy" OR "painting" OR "drawing")
AND
("cognitive dysfunction" OR "cognitive impairment" OR "mild cognitive impairment" OR "mild cognitive decline" OR "MCI" OR "subjective cognitive complaint" OR "subjective memory impairment")
AND
("elderly" OR "older adults" OR "aged" OR "geriatric")

3. **Cochrane Library**

("art therapy" OR "visual art therapy" OR "visual art" OR "visual aesthetic*" OR "art intervention*" OR "creative art*" OR "art psychotherapy" OR painting OR drawing):ti,ab,kw
AND
("cognitive dysfunction" OR "cognitive impairment" OR "mild cognitive impairment" OR "mild cognitive decline" OR MCI OR "subjective cognitive complaint*" OR "subjective memory impairment"):ti,ab,kw
AND
(elderly OR "older adult*" OR aged OR geriatric):ti,ab,kw

4. **Web of Science**

TS=("art therapy" OR "visual art therapy" OR "visual art" OR "visual aesthetic" OR "art intervention" OR "creative art" OR "art psychotherapy" OR "painting" OR "drawing")
AND
TS=("cognitive dysfunction" OR "cognitive impairment" OR "mild cognitive impairment" OR "mild cognitive decline" OR "MCI" OR "subjective cognitive complaint" OR "subjective memory impairment")
AND
TS=("elderly" OR "older adults" OR "aged" OR "geriatric")

5. **Embase**

('art therapy' OR 'visual art therapy' OR 'visual art' OR 'visual aesthetic' OR 'art intervention' OR 'creative art' OR 'art psychotherapy' OR 'painting' OR 'drawing')
AND
('cognitive dysfunction' OR 'cognitive impairment' OR 'mild cognitive impairment' OR 'mild cognitive decline' OR 'MCI' OR 'subjective cognitive complaint' OR 'subjective memory impairment')
AND
('elderly' OR 'older adults' OR 'aged' OR 'geriatric')

6. **Scopus**

TITLE-ABS-KEY("art therapy" OR "visual art therapy" OR "visual art" OR "visual aesthetic" OR "art intervention" OR "creative art" OR "art psychotherapy" OR "painting" OR "drawing")
AND
TITLE-ABS-KEY("cognitive dysfunction" OR "cognitive impairment" OR "mild cognitive impairment" OR "mild cognitive decline" OR "MCI" OR "subjective cognitive complaint" OR "subjective memory impairment")
AND
TITLE-ABS-KEY("elderly" OR "older adults" OR "aged" OR "geriatric")

7. **CINAHL**

("art therapy" OR "visual art therapy" OR "visual art" OR "visual aesthetic" OR "art intervention" OR "creative art" OR "art psychotherapy" OR "painting" OR "drawing")
AND
("cognitive dysfunction" OR "cognitive impairment" OR "mild cognitive impairment" OR "mild cognitive decline" OR "MCI" OR "subjective cognitive complaint" OR "subjective memory impairment")
AND
("elderly" OR "older adults" OR "aged" OR "geriatric")

8. **ProQuest**

("art therapy" OR "visual art therapy" OR "visual art" OR "visual aesthetic" OR "art intervention" OR "creative art" OR "art psychotherapy" OR "painting" OR "drawing")
AND
("cognitive dysfunction" OR "cognitive impairment" OR "mild cognitive impairment" OR "mild cognitive decline" OR "MCI" OR "subjective cognitive complaint" OR "subjective memory impairment")
AND
("elderly" OR "older adults" OR "aged" OR "geriatric")

9. **CNKI**

AB=("艺术治疗"+"美术治疗"+"视觉艺术"+"艺术干预"+"创造性艺术"+"绘画"+"拼贴画"+"雕塑")
AND
AB=("认知障碍"+"认知损害"+"认知衰退"+"认知下降"+"MCI"+"主观记忆")
AND

AB=("老年"+"年长"+"高龄")

10. ClinicalTrials.gov

AREA[ConditionSearch]("cognitive impairment" OR "cognitive decline" OR "memory impairment")
AND AREA[BasicSearch]("art therapy" OR "visual art" OR painting OR drawing OR collage OR crafts)
AND AREA[StudyType](INTERVENTIONAL)

11. ProQuest Dissertations & Theses Global

noft(("visual art therapy" OR "art therapy" OR "visual art" OR "art intervention" OR "art psychotherapy" OR painting OR drawing OR collage OR calligraphy OR sculpture OR pottery OR "arts and crafts" OR "art appreciation"))
AND
abstract(("mild cognitive impairment" OR MCI OR "cognitive impairment" OR "cognitive decline" OR "mild neurocognitive disorder" OR "subjective cognitive decline" OR "subjective memory impairment"))
AND
abstract((elderly OR "older adult*" OR aged OR geriatric OR senior*))

12. WHO ICTRP

Condition：mild cognitive impairment OR cognitive impairment OR cognitive decline OR memory impairment
Intervention：art therapy OR visual art OR painting OR drawing OR collage OR crafts OR calligraphy
