# Supplementary material for: The effects of visual art therapy in older adults with mild cognitive impairment: a systematic review and meta-analysis
Source: Front Public Health. 2026 Mar 23;14:1765620. doi: 10.3389/fpubh.2026.1765620 (PMC13051655; doi:10.3389/fpubh.2026.1765620)
Supplement: Supplementary file 3 [file Data_Sheet_3.pdf]

Sensitivity analyses for pre–post correlation and subgroup analyses

1. Sensitivity analyses for pre–post correlation

When SD_{change} was not reported, SD_{change} was imputed from baseline and post-intervention SDs using an assumed within-group pre-post correlation (*r*). Primary analyses used *r*=0.50. Sensitivity analyses repeated all meta-analyses with *r*=0.25 and *r*=0.75. The sensitivity analysis results for the primary and secondary outcomes are presented below.

$$SD_{change}=\sqrt{SD_{baseline}^{2}+SD_{post}^{2}-2r\cdot SD_{baseline}\cdot SD_{post}}$$

1.1 Assuming pre-post correlation *r*=0.25

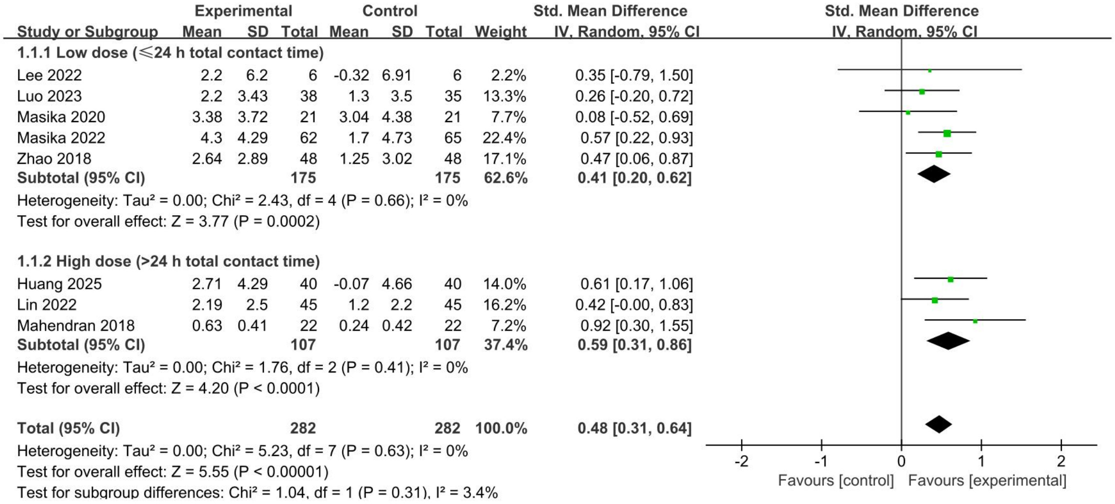

Figure S1a Impact of VAT on global cognitive performance (MoCA).

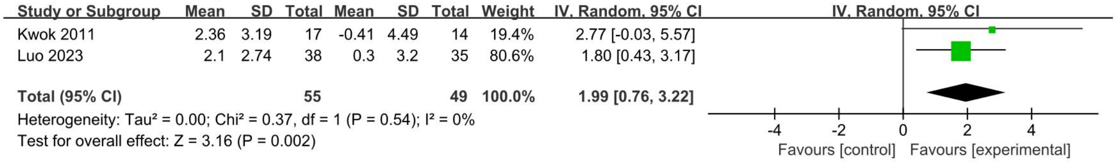

Figure S2a Impact of VAT on global cognitive performance (MMSE).

Study or Subgroup	Experimental Mean	Experimental SD	Experimental Total	Control Mean	Control SD	Control Total	Weight	Std. Mean Difference IV, Random, 95% CI
**2.2.1 AVLT-DR**								
Kwok 2011	0.43	1.14	17	0	1.19	14	4.6%	0.36 [-0.35, 1.07]
Lin 2022	2.11	1.6	45	1.02	2.84	45	13.4%	0.47 [0.05, 0.89]
Luo 2023	2	3.67	38	1.3	3.5	35	11.1%	0.19 [-0.27, 0.65]
Mahendran 2018	0.31	0.47	22	0.08	0.47	22	6.5%	0.48 [-0.12, 1.08]
Zhao 2018	3.03	3.79	48	2.27	4.66	45	14.2%	0.18 [-0.23, 0.59]
**Subtotal (95% CI)**			**170**			**161**	**49.8%**	**0.32 [0.10, 0.53]**
Heterogeneity: Tau² = 0.00; Chi² = 1.53, df = 4 (P = 0.82); I² = 0%								
Test for overall effect: Z = 2.85 (P = 0.004)								
**2.2.2 AVLT-IR**								
Huang 2025	2.74	7.22	40	1.34	5.02	40	12.2%	0.22 [-0.22, 0.66]
Lin 2022	3.79	6.4	45	1.68	3.7	45	13.5%	0.40 [-0.02, 0.82]
Luo 2023	2.3	7.44	38	1	5.88	35	11.1%	0.19 [-0.27, 0.65]
Zhao 2018	1.76	0.69	48	1.32	0.7	45	13.5%	0.63 [0.21, 1.05]
**Subtotal (95% CI)**			**171**			**165**	**50.2%**	**0.37 [0.16, 0.59]**
Heterogeneity: Tau² = 0.00; Chi² = 2.50, df = 3 (P = 0.48); I² = 0%								
Test for overall effect: Z = 3.37 (P = 0.0007)								
**Total (95% CI)**			**341**			**326**	**100.0%**	**0.34 [0.19, 0.50]**
Heterogeneity: Tau² = 0.00; Chi² = 4.16, df = 8 (P = 0.84); I² = 0%								
Test for overall effect: Z = 4.40 (P < 0.0001)								
Test for subgroup differences: Chi² = 0.13, df = 1 (P = 0.72), I² = 0%								

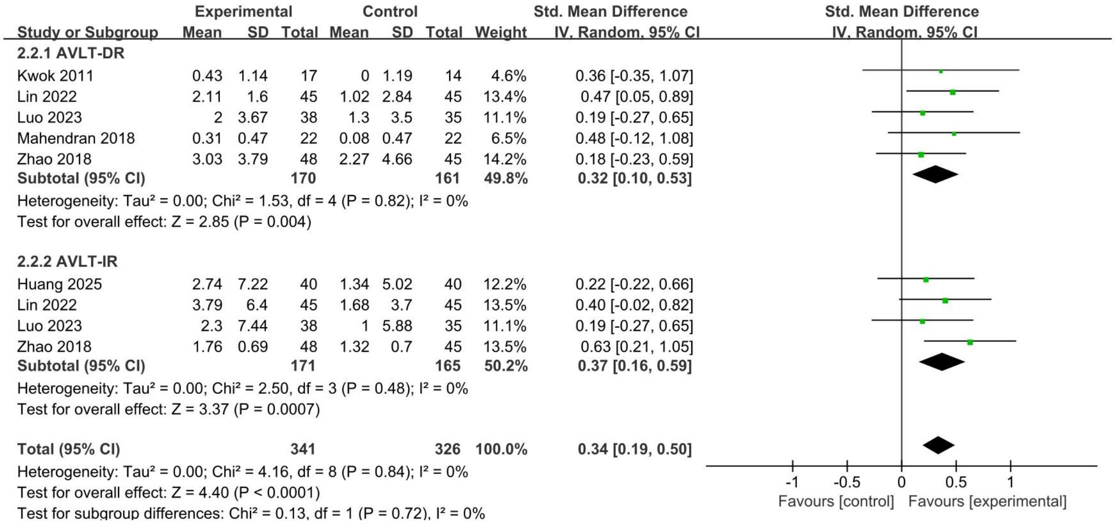

Figure S3a Impact of VAT on memory function.

Study or Subgroup	Experimental Mean	Experimental SD	Experimental Total	Control Mean	Control SD	Control Total	Weight	Std. Mean Difference IV, Random, 95% CI
Huang 2025	0.94	4.05	40	0.96	3.77	40	29.0%	-0.01 [-0.44, 0.43]
Kwok 2011	0.14	1.31	17	0	1.21	14	11.1%	0.11 [-0.60, 0.82]
Luo 2023	0.3	4.47	38	-0.6	4.96	35	26.3%	0.19 [-0.27, 0.65]
Zhao 2018	1.59	3.99	48	0.9	4.35	45	33.6%	0.16 [-0.24, 0.57]
**Total (95% CI)**			**143**			**134**	**100.0%**	**0.12 [-0.12, 0.35]**
Heterogeneity: Tau² = 0.00; Chi² = 0.44, df = 3 (P = 0.93); I² = 0%								
Test for overall effect: Z = 0.96 (P = 0.34)								

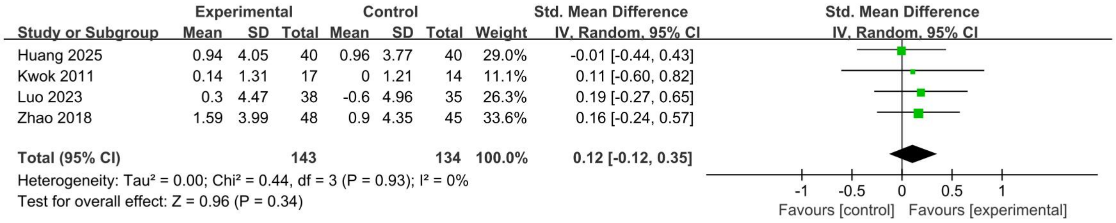

Figure S4a Impact of VAT on language function.

Study or Subgroup	Experimental Mean	Experimental SD	Experimental Total	Control Mean	Control SD	Control Total	Weight	Std. Mean Difference IV, Random, 95% CI
Lee 2022	-1.9	3.5	6	-0.43	5.48	6	4.3%	-0.30 [-1.44, 0.85]
Lin 2022	-1.28	2.76	45	-0.4	3.2	45	29.5%	-0.29 [-0.71, 0.12]
Mahendran 2018	-1.06	2.03	22	-0.47	2.11	22	15.1%	-0.28 [-0.87, 0.31]
Masika 2020	-3	2.78	21	0	2.66	18	11.7%	-1.08 [-1.76, -0.40]
Masika 2022	-3.03	2.67	62	-1.7	2.94	65	39.4%	-0.47 [-0.82, -0.12]
**Total (95% CI)**			**156**			**156**	**100.0%**	**-0.45 [-0.69, -0.22]**
Heterogeneity: Tau² = 0.00; Chi² = 4.25, df = 4 (P = 0.37); I² = 6%								
Test for overall effect: Z = 3.75 (P = 0.0002)								

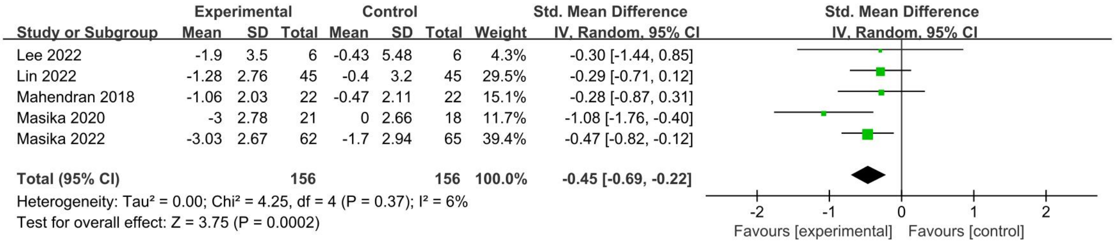

Figure S5a Impact of VAT on depressive symptoms.

Study or Subgroup	Experimental Mean	Experimental SD	Experimental Total	Control Mean	Control SD	Control Total	Weight	Std. Mean Difference IV, Random, 95% CI
Lee 2022	-0.08	0.46	6	0.01	0.57	6	4.0%	-0.16 [-1.29, 0.97]
Luo 2023	-0.3	0.89	38	-0.1	0.75	35	24.1%	-0.24 [-0.70, 0.22]
Masika 2022	-0.8	1.55	62	-0.3	1.46	65	41.8%	-0.33 [-0.68, 0.02]
Zhao 2018	-2.31	3.79	48	-0.57	3.3	45	30.1%	-0.48 [-0.90, -0.07]
**Total (95% CI)**			**154**			**151**	**100.0%**	**-0.35 [-0.57, -0.12]**
Heterogeneity: Tau² = 0.00; Chi² = 0.75, df = 3 (P = 0.86); I² = 0%								
Test for overall effect: Z = 3.01 (P = 0.003)								

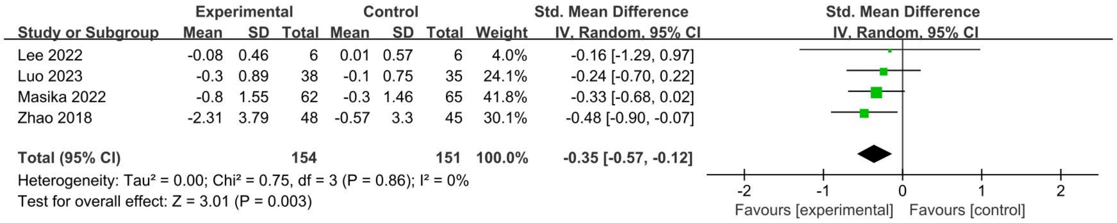

Figure S6a Impact of VAT on activities of daily living.

1.2 Assuming pre-post correlation r=0.75

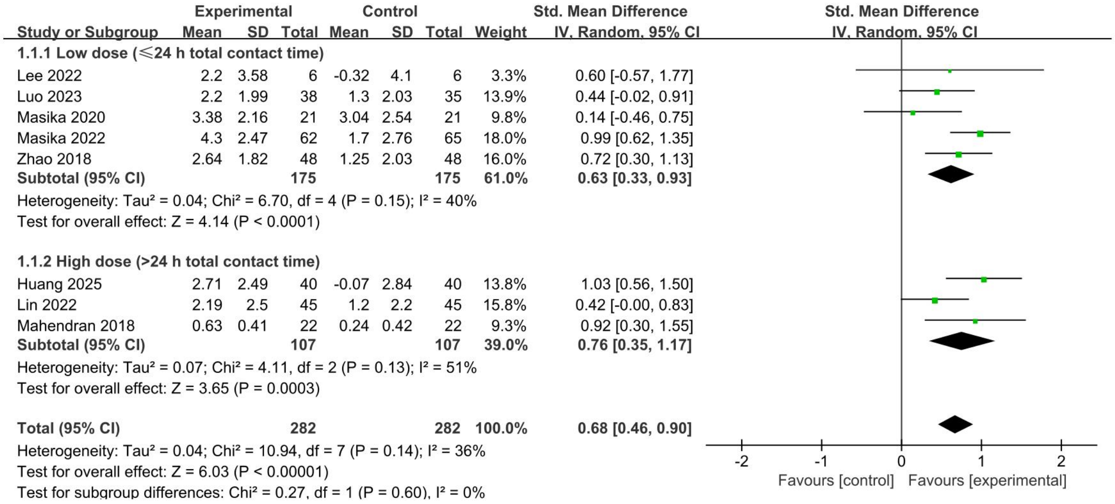

Figure S1b Impact of VAT on global cognitive performance (MoCA).

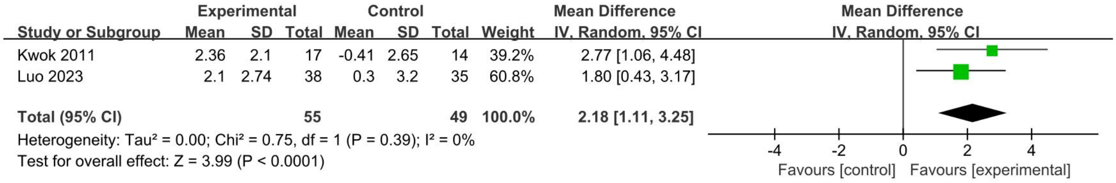

Figure S2b Impact of VAT on global cognitive performance (MMSE).

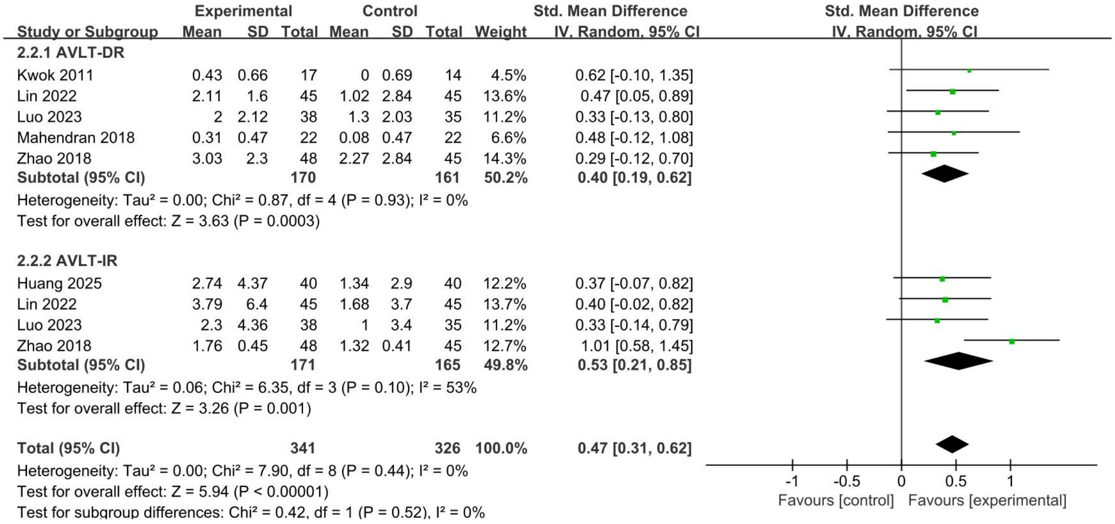

Figure S3b Impact of VAT on memory function.

Study or Subgroup	Experimental Mean	Experimental SD	Experimental Total	Control Mean	Control SD	Control Total	Weight	Std. Mean Difference IV, Random, 95% CI
Huang 2025	0.94	2.34	40	0.96	2.26	40	29.1%	-0.01 [-0.45, 0.43]
Kwok 2011	0.14	0.76	17	0	0.7	14	11.1%	0.19 [-0.52, 0.89]
Luo 2023	0.3	2.79	38	-0.6	2.87	35	26.2%	0.31 [-0.15, 0.78]
Zhao 2018	1.59	2.88	48	0.9	2.98	45	33.6%	0.23 [-0.17, 0.64]
**Total (95% CI)**			**143**			**134**	**100.0%**	**0.18 [-0.06, 0.42]**

Heterogeneity: Tau² = 0.00; Chi² = 1.10, df = 3 (P = 0.78); I² = 0%
Test for overall effect: Z = 1.48 (P = 0.14)

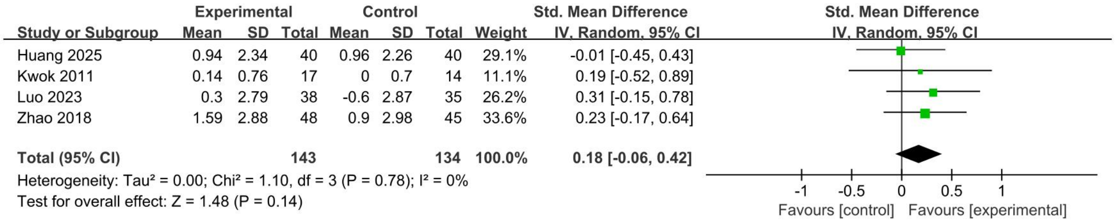

Figure S4b Impact of VAT on language function.

Study or Subgroup	Experimental Mean	Experimental SD	Experimental Total	Control Mean	Control SD	Control Total	Weight	Std. Mean Difference IV, Random, 95% CI
Lee 2022	-1.9	2.07	6	-0.43	3.2	6	10.9%	-0.50 [-1.66, 0.65]
Lin 2022	-1.28	2.76	45	-0.4	3.2	45	24.9%	-0.29 [-0.71, 0.12]
Mahendran 2018	-1.06	2.03	22	-0.47	2.11	22	20.8%	-0.28 [-0.87, 0.31]
Masika 2020	-3	1.61	21	0	1.6	18	17.2%	-1.83 [-2.59, -1.07]
Masika 2022	-3.03	1.88	62	-1.7	1.7	65	26.1%	-0.74 [-1.10, -0.38]
**Total (95% CI)**			**156**			**156**	**100.0%**	**-0.69 [-1.17, -0.22]**

Heterogeneity: Tau² = 0.19; Chi² = 13.82, df = 4 (P = 0.008); I² = 71%
Test for overall effect: Z = 2.85 (P = 0.004)

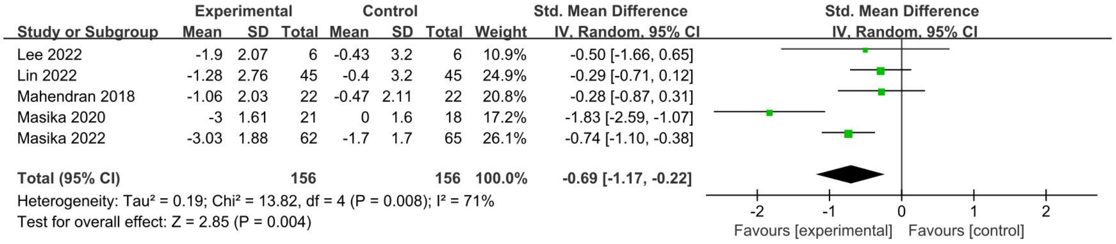

Figure S5b Impact of VAT on depressive symptoms.

Study or Subgroup	Experimental Mean	Experimental SD	Experimental Total	Control Mean	Control SD	Control Total	Weight	Std. Mean Difference IV, Random, 95% CI
Lee 2022	-0.08	0.26	6	0.01	0.37	6	4.1%	-0.26 [-1.40, 0.88]
Luo 2023	-0.3	0.66	38	-0.1	0.46	35	24.5%	-0.35 [-0.81, 0.12]
Masika 2022	-0.8	0.93	62	-0.3	0.94	65	41.9%	-0.53 [-0.89, -0.18]
Zhao 2018	-2.31	2.4	48	-0.57	2.11	45	29.5%	-0.76 [-1.18, -0.34]
**Total (95% CI)**			**154**			**151**	**100.0%**	**-0.54 [-0.77, -0.31]**

Heterogeneity: Tau² = 0.00; Chi² = 1.98, df = 3 (P = 0.58); I² = 0%
Test for overall effect: Z = 4.64 (P < 0.00001)

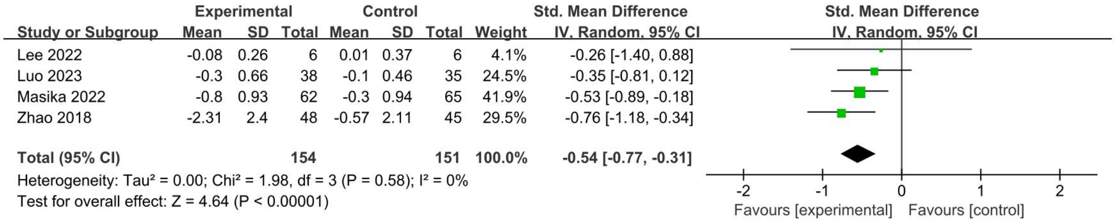

Figure S6b Impact of VAT on activities of daily living.

2. Subgroup analyses

Study or Subgroup	Experimental Mean	Experimental SD	Experimental Total	Control Mean	Control SD	Control Total	Weight	Std. Mean Difference IV, Random, 95% CI
**1.1.1 Passive control**								
Huang 2025	2.71	3.51	40	-0.07	3.86	40	13.8%	0.75 [0.29, 1.20]
Lee 2022	2.2	5.07	6	-0.32	5.68	6	2.2%	0.43 [-0.72, 1.58]
Lin 2022	2.19	2.5	45	1.2	2.2	45	16.3%	0.42 [-0.00, 0.83]
Mahendran 2018	0.63	0.41	22	0.24	0.42	22	7.3%	0.92 [0.30, 1.55]
**Subtotal (95% CI)**			**113**			**113**	**39.6%**	**0.63 [0.36, 0.89]**
Heterogeneity: Tau² = 0.00; Chi² = 2.21, df = 3 (P = 0.53); I² = 0%								
Test for overall effect: Z = 4.57 (P < 0.00001)								
**1.1.2 Active control**								
Luo 2023	2.2	2.81	38	1.3	2.86	35	13.3%	0.31 [-0.15, 0.78]
Masika 2020	3.38	3.04	21	3.04	3.58	21	7.8%	0.10 [-0.50, 0.71]
Masika 2022	4.3	3.5	62	1.7	3.87	65	22.1%	0.70 [0.34, 1.06]
Zhao 2018	2.64	2.41	48	1.25	2.57	48	17.1%	0.55 [0.15, 0.96]
**Subtotal (95% CI)**			**169**			**169**	**60.4%**	**0.49 [0.25, 0.72]**
Heterogeneity: Tau² = 0.01; Chi² = 3.55, df = 3 (P = 0.31); I² = 15%								
Test for overall effect: Z = 3.98 (P < 0.0001)								
**Total (95% CI)**			**282**			**282**	**100.0%**	**0.55 [0.38, 0.72]**

Heterogeneity: Tau² = 0.00; Chi² = 6.30, df = 7 (P = 0.50); I² = 0%
Test for overall effect: Z = 6.36 (P < 0.00001)
Test for subgroup differences: Chi² = 0.59, df = 1 (P = 0.44), I² = 0%

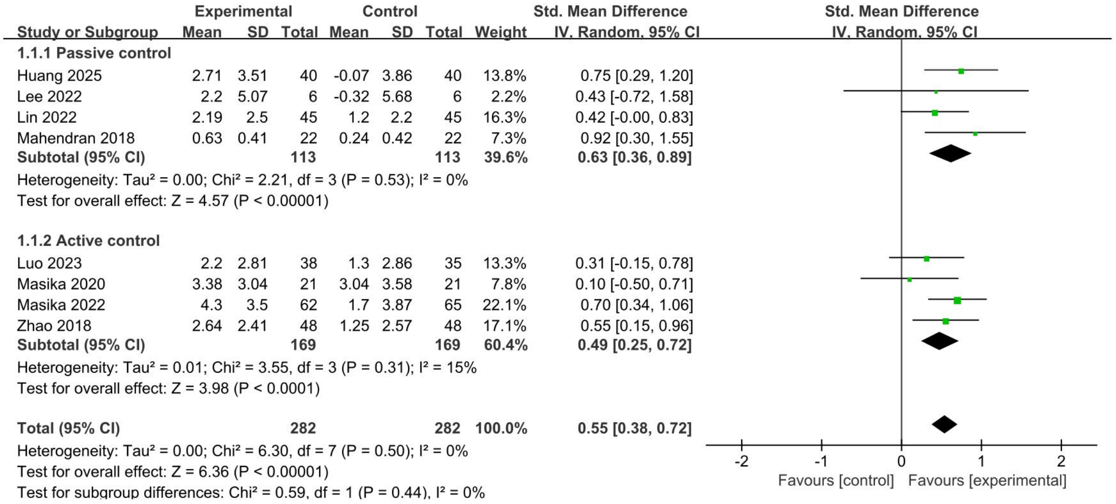

Figure S1c Impact of VAT on global cognitive performance (MoCA).
